# Flooding the Urinary Tract1Read at the regular meeting of the Chemung County Medical Society, at Elmira, August 20, 1895.

**Published:** 1895-10

**Authors:** B. H. Daggett

**Affiliations:** Buffalo, N. Y.


### FLOODING THE URINARY TRACT.[1]

BY B. H. DAGGETT, M. D., Buffalo, N. Y.

IN RESPONSE to your flattering invitation, I am here today to describe and demonstrate a new method of irrigating the bladder without a catheter and without force.

In a paper read before the American Association of Genito-urinary Surgeons, at Washington, June 1, 1894, Dr. G. E. Brewer states that Dr. C. B. Crossfield, in 1792, contributed an article to the *New London Medical Journal*, in which he reported success in irrigating the bladder from the urethral meatus, by means of continuous pressure applied by an ordinary bulb syringe.

In an article contributed to the *Virginia Medical Monthly*, in 1877, Dr. Hunter McGuire reported successful irrigation of the bladder without a catheter, by a similar procedure.

Dr. Brewer states that his attention was called, about three years before, to a report of some experiments by H. Felike, of Budapest, in which Felike demonstrated that the resistance of the urethral compressor muscle can be overcome by the pressure of a column of water from one to three meters in height. Felike places his patients in a horizontal position on a bed or a table. The bladder having been emptied, the nozzle of the syringe is inserted and compressed in the urethral orifice, the fountain is raised to the height of two meters, when the patient is instructed to take one or more deep and rapid inspirations. I quote Dr. Brewer, who recently witnessed this procedure at the clinic of Dr. Felike: "In a few minutes the subject of the experiment would express a desire to urinate, when it would usually be found that the bladder had been completely filled, without the slightest discomfort to the patient and often without his knowledge." If this statement be exact, the foreign bladder differs remarkably from the Yankee organ. I have seldom found that the bladder could be filled com-

---

1. Read at the regular meeting of the Chemung County Medical Society, at Elmira, August 20, 1895.

pletely before tolerance is established by frequent irrigations, at least in younger men. The majority of patients complain of vesical tenesmus after three or four ounces have passed into the viscus, and insist upon urination. Dilatation of the bladder by hot saline solutions causes, sooner or later, rhythmic movements and clonic contractions.

Any fluid passed into the bladder, whether by force or otherwise, will cause tenesmus and an urgent desire to urinate, long before the bladder is filled, in a majority of cases. The bladder of the hypertrophic prostatic is more tolerant, however, and, in some cases, may be well filled from the first.

The younger men usually feel the passing of the fluid through the deep urethra, and after having once experienced this feeling, they realise that they have acquired the knack of flushing the bladder at will.

In the case of the chronic hypertrophic prostatic, the deep urethra and bladder are less sensitive and only give warning of the passing of the fluid by a desire to urinate after more or less has accumulated in this viscus.

Keys, in the *Annual of the Universal Medical Sciences*, Vol. III., 1892, refers to his experiments with the method of Rona of forcing medicated fluids into the deep urethra for the relief of posterior urethritis, and says his results were poor.

Lydston used forced injections by a bulb syringe, while the patient was straining as if to urinate, and said that the results were not satisfactory.

Janet employed the method proposed by Felike and condemned it, saying that it did harm in cystitis. One can readily believe that the force of a column of water from six to twelve feet in height, producing hydrostatic pressure of from three and one-half to six pounds per square inch, would be traumatically harmful to an inflamed bladder, and scarcely endurable in the normal viscus. The practice of Guion, at the Neckar Hospital, is similar to that of Felike—namely, the use of hydrostatic force, the patient being in the horizontal position, struggling in the throes of forced and frequent inspirations, and possesses no advantage over that other plan of forced injections with a bulb syringe during vesical strain.

These methods of force overcome or tire out the urethral sphincters, and in this way the fluid passes into the bladder with more or less violence. The length of time required for tiring out these muscles varies greatly in different cases and in the same case at different times. These methods often fail entirely in overcoming the resistance of the muscles. The pressure of the water is about half a pound to the square inch for each foot of height—to be more accurate, four and three-fourths pounds for a ten-foot column of water at a temperature of 110 degrees.

From six to ten feet is the height of the volume recommended by Felike. One can readily believe that the impact of a stream of water, under the pressure of three to five pounds of force, passing in jets against the walls of the inflamed viscus, would aggravate and not relieve, and the same reasoning would apply to forced injections by a bulb syringe. For these reasons, probably, the profession has not adopted these methods.

At the regular meeting of the Lake Erie Medical Association, July 15, 1892, I read an article describing a new method of irrigating the deep urethra and bladder without a catheter. I will again describe the technique of this process since doubts of its practicability have been expressed and failures have been reported.

The materials are a three or four quart fountain, five or six feet of rubber tubing, with a shut-off placed near its distal end. The irrigant is water at a temperature of 110 or 112 degrees Fahr., rendered bland by the solution of a little salt, soda, potash, mucilage or some viscid, diffusible substance. The fountain is raised about a foot above the plane of the pelvis and the tube connected with the inlet of the irrigator. Posture is an essential factor in carrying out this procedure. The patient must assume a semi-dorsal position, with the trunk at an angle of about forty-five degrees with the horizontal line. The thighs should be flexed upon the pelvis, thus giving the body a reversed squatting position. This posture gives an almost directly downward course to the fixed urethra. These flexures of the body relax the muscles of the pelvic floor, which in turn, by direct connection, relieve tension of the urethral sphincter. This posture gives the most favorable condition for inducing vermicular rhythmical action of the urethral canal, when a continuous flow of a hot saline fluid at an even temperature, greater than that of the body, is applied.

Civiale observed and noted that the urethral canal would take on a swallowing action.

Rawdon McNamarra, in an article contributed to the *London Medical Press and Circular*, about 1872, maintained that there was a vermicular action bladderward of the urethra. With the patient placed upon the back in a semi-recumbent posture, the limbs flexed and supported in order to avoid tension, I have noted that instruments would pass the urethra without force, in a regular, gentle, yet quite perceptible hitching motion. This is especially noted when using the bulbous bougie and is more apparent if the rigid instead of the flexible instrument be used.

There would appear to be a ciliary or vibratory motion of the epithelium, or it may be a vermicular motion of the muscles which causes apparently a suction action of the urethral canal, and which probably aids in the retention of the urine. This property of the urethra, whether it be ciliary, vermicular or peristaltic, is especially noticeable in the deep urethra.

The claim that force is not necessary for introducing water into the bladder without a catheter is further demonstrated by the history of the following case :

A man upon whom I had performed an external urethrotomy (perineal section), passed water into his bladder by this method, with the false passage still open, so that in urination about half of this stream passed through the urethral canal, the balance escaping through this false passage. It is evident that this fluid did not gain entrance into the bladder by force, as the fountain was placed but twelve or fourteen inches higher than the position of the bladder, so that the column of water would bear the hydrostatic pressure of less than half a pound to the square inch, which would be lessened by the friction of the water passing through six feet of tubing, further reduced by the outflow in the canula, and still further reduced by the false passage resulting from the urethrotomy which, as stated above, was sufficiently open to draw off about half of the stream in micturition. These two outlets exceed the inlet in bulk, still he passed one-half pint into the bladder.

The fact that water may be passed into the bladder without force is further demonstrated by the following case :

A man 84 years of age, who had not passed a drop of water by normal urination for more than seven months, was brought before me as a test case. My assistant readily passed, by this coaxing method, sixteen ounces of water into the bladder. His inability to urinate was caused by an enlarged prostate.

I have further tested the necessity of force, continuous or otherwise, by closely pushing the wedge-shaped canula into the urethral meatus, where it would be held without compressing the penile organ, without displacement and without urethral leakage, the pendulous penis being held in line with the fixed urethra, and in this way passed fluid into the bladder.

This case illustrates the fact, well known to genito-urinary surgeons, that the bladder of the hypertrophic prostatic is more tolerant than the normal viscus. Its rhythmic action was destroyed; at least it did not respond to dilatation and the presence of the hot saline solution.

It is well known that all the tubular and visceral organs of the body manifest a rhythmic action. The sphincteric movements are tonic and are due to the action of the muscular fibers through their special ganglia.

In an article contributed to the *Medical Bulletin* of Philadelphia for August, 1895, Dr. Ott says, as a result of his experiments, that weak saline solutions over a temperature of seventy degrees Fahr., cause a series of contractions which continue for several hours, in the distended bladder of a cat, and that heat increases these movements; they also continue after the division and destruction of the spinal cord in the lumbar region. Galvanism, directly applied, causes prolonged tonic contractions.

Various remedies were used, both locally and generally, to increase its rhythmic contraction. Ergotin and potash were the most active—potash produced a powerful action on the bladder, even greater than ergot. Ott states: "Hence, in commencing hypertrophy of the prostate, a combination of the soluble potash salts will be a great improvement over the ergot treatment alone."

Ott refers to Sokownin's discovery that the inferior mesenteric ganglion is the seat of reflexes for the bladder, and concludes from his own experiments that the vesical rhythmic movements are due to extra- or intravesical ganglia acting upon the unstriped muscle, and perhaps to the involuntary muscular fiber and to the intravesical ganglia.

Rhythmic action of the urethra is promoted or induced by hot saline irrigation and rhythmic movements are also induced in the bladder after more or less distention by the fluid, so that the rhythmic action of the urethra is followed by that of the bladder after it is distended.

Twenty years ago Gouley experimented with bladder gymnastics, and reported that his results were not satisfactory. His purpose was to dilate contracted bladders by forced injections.

In the *Medical Record* for April 13, 1895, Dr. Fuller, treating of perivesical inflammation and resulting adhesions, says:

For local treatment, bladder gymnastics, so-called, (Daggett, of Buffalo, has written on this form of treatment,) seem to be of some benefit, though the good derived thereby is generally but temporary. This form of treatment consists of the regular stretching of the bladder walls by the injection into it, under regulated pressure, of a warm, antiseptic fluid.

Doctors Gouley and Fuller attempted stretching of the contracted bladder by force. My proposition was to gently exercise atonic or dilated bladders by passing aseptic fluids without force, to be expelled by normal urination, repeating this process two or three times daily, flushing the bladder three or four times at each séance, as illustrated in Case III. This is a gentle method, continuously exercising the vesical muscular structures for the relief of atonic conditions, and enabling the bladder to promptly and completely expel its contents. I have noted on two occasions, during discussions in the New York Academy of Medicine, reference to the catheterless bladder irrigation, in which the opinion prevailed that the urethral constrictors must be relaxed by continuous force.

Dr. Bauer, of St. Louis, in an article printed in the *Charlotte Medical Journal*, says, referring to posterior urethritis, "so long as a drop of urine is allowed to pass the urethra with pathological force, so long will it be impossible to effectually cure these cases." If pathological force in voiding the urine is harmful, certainly reversing this process and using hydrostatic pressure, or the force of a bulb syringe, during vesical strain must be still more injurious.

The younger men note the passing of the water through the deep urethra, unless it has been made insensitive by instrumentation or chronic disease, but older men do not have this sensation and are warned by the presence of the water in the bladder after it has accumulated in a considerable quantity, causing a desire to urinate, unless the fluid be very warm, when the heat is felt as the water accumulates in that organ.

A suitable device for maintaining this position may be extemporised in any household by using a hip bath, an ordinary bath tub, a rocking or ordinary chair, tilted and blocked, with the lower extremities placed upon a foot rest, or bed, or any convenient support.

If the urethra be permeable, strict compliance with these details will insure success in passing water into any bladder without force, without violence and without a catheter.

As soon as tenesmus is felt, the bladder should be emptied by normal urination, which may be done without changing position, and this procedure repeated until the water comes clear. There is no force or violence to induce traumatism, and no threat of infection from a catheter. This is really a coaxing process readily learned and applied by the patient himself, and as it brings comfort and relief it is faithfully employed. It is a curious fact that laymen, under verbal or written instructions, readily and successfully apply this method in their own cases, while physicians, owing to preconceived notions or prejudice, often fail in its application.

This plan of irrigation is especially valuable for the treatment of cystitis as well as inflammatory affections of the adnexa of the urinary tract.

It is especially valuable when the inflammation involves the cord and epididymis, conditions in which catheterisation and forced injections are contra-indicated. The following cases will illustrate this point :

Case I.—P., aged 30 years, had been confined to his room seven and one-half weeks suffering from inflammation of the bladder and posterior urethra and recurring attacks of epididymitis. Irrigation by catheter was employed daily to relieve the urgent symptoms and if it was neglected the temperature of the body would rise. Catheter irrigation kept the inflammation alive. P. was taught the lesson of auto-irrigation and flooded the lower urinary tract by this method. Relief was decided and prompt, and after three days of this treatment he was a convalescent and suffered no relapse. P. had been under the care of a skilful surgeon of Buffalo for nearly two months, who had employed the best known methods of treatment, and it is evident that he could not have been relieved by catheter irrigation. Without the use of this coaxing process a surgical procedure would have been necessary.

Case II.—H. had suffered from recurring attacks of epididymo-orchiditis, its predisposing cause being a chronic posterior urethritis, the result of gonorrhea. These attacks were alleged to have been induced by unusual physical exertion—excess in diet, or venery. The predisposing cause was constant—namely, a chronic posterior urethritis. Irritant urethral injections had been used which may have acted as an exciting cause of these recurrences. Flushing of the lower urinary tract forestalled any return.

Case III.—R., 63 years of age, gives the following history of his sufferings : In a mining camp, twenty-three years ago, he had a very severe attack of cystitis caused by drinking alkaline water. He came home on this account, was ill several months and never fully recovered. He had been confined to his room four weeks, drugs failing to bring relief; irrigation by double catheter was employed. Still his condition grew steadily worse. His attending physician, realising that a crisis was at hand, proposed to call in a surgeon to do cystotomy. R. declined this service and called me to take charge of his case. At this time he presented all the phenomena of septic infection. His urine was strongly alkaline, offensive, depositing one-quarter part, by volume, solid matter, consisting of pus and inflammatory débris. R. readily learned self-irrigation without the catheter and cleared his urine in five days, and was able to attend his office. There still remained a tendency to relapse, which was controlled by irrigation.

It was noted that irrigations not only warded off this tendency, but they also relieved soreness and pain, as he expressed himself were luxurious and enjoyed three times each day. R. would completely empty his bladder as he supposed. The first washing would show straw or amber color, the third would be clear. He passed into his bladder half a pint of hot milk, and maintained that as hot milk was good for sore eyes, therefore it ought to be good for sore bladders. After micturating, he irrigated, and the first washing was very milky, the third showed up clear. These tests indicated residuum which, becoming disturbed, caused cystitis. The prostate is not perceptibly enlarged and he has never had retention. He firmly refused permission to pass the catheter to test the question of residual urine, alleging that he had already suffered sufficiently from the use of that instrument for irrigation.

After doing bladder gymnastics by three daily irrigations for six weeks, it is evident that this viscus is completely evacuated by normal urination, and more than this, the rising stream is ejected with sufficient force to menace facial autonomy. Gymnastics of the lower urinary apparatus had relieved urinary stasis and its ever attending threat.

Case IV.—B., 20 years old, a railroad fireman, had been confined to his bed five days suffering from prostato-cystitis, caused by gonorrhea, passing thick decomposing urine every ten to fifteen minutes. He succeeded in the second trial in flushing the bladder. The swelling of a prostatic abscess interrupted or blocked deep irrigation for eighteen hours. Immediately following the rupture of the abscess, irrigation was successful and uninterrupted to the end. Pain practically ceased in three days and within a week he could hold his water for six hours. For three days following the rupture he passed masses of macerated blood coagula, which was made possible by flooding the bladder and evacuating its contents by normal urination. Recovery was speedy and complete.

Case V.—W., 61 years old, a house painter, states that for five years his urine has passed away by dribbling, constantly necessitating the use of protectives. April 20, 1894, he had a severe attack of strangury, and his physician being unable after repeated efforts to pass a catheter, palliated the symptoms by the administration of drugs. W.

continued to suffer from distention and tenesmus. Dr. Stuart was called April 28th, and after making diligent efforts failed to pass a catheter. The case becoming urgent, Dr. Stuart says : "I determined to test the method proposed by Dr. Daggett—namely, relaxation by posture and hot water irrigation. My patient was seated in a low rocking chair, tilted as described in this method, and his legs placed on the tops of two other chairs. The canula was introduced and within a short time he expressed a desire to urinate. The canula was then removed and he passed nearly a pint of fluid. Irrigation was used twice daily for a week, then once daily since that time. Convalescence has been uninterrupted, and his power to void urine has steadily improved. At present W. has no difficulty in urinating, dribbling has ceased and he feels better than for several years past."

Case VI.—B., 34 years of age, married, consulted me June 28th, stating that he had suffered from inflammation of the urinary tract for nearly two months and had been surfeited with injections and catheter irrigation, that in spite of or in consequence of this treatment the inflammatory affection had extended and involved the left cord and testicle. B. gave a history of urethritis followed by cystitis. At this time he had prostato-cystitis and this inflammatory condition had extended to the left testicle. B. stated that he had been confined to his house three weeks and that his condition had been growing worse. He was instructed to get an irrigating apparatus and begin its use at once. Under this treatment the inflammation steadily subsided and in four days B. was enabled to return to his work. Irrigation, in a week's time, had reduced the inflammatory action so that local treatment by powder insufflation was instituted. B. writes August 5th : "I believe that I am a well man. There is no urethral discharge, the urine is clear and there are no symptoms remaining, thanks to your method or treatment."

### IRRIGATION IN CASE OF CHRONIC HYPERTROPHIC PROSTATE.

It is stated that about 33½ per cent. of men between the ages of fifty-five and sixty have more or less enlargement of the prostate, and a small percentage of these cases suffer from urinary obstruction and its attendant evils.

Enlargement of the prostate may delay, but will not prohibit passing a fluid into the bladder by this process. I have succeeded in passing water into the bladder in cases where the patient was unable to void it without a catheter. I will relate the following case for the purpose of illustrating the use of this method of irrigation in treatment of enlarged prostate.

Case VII.—A. S., 74 years of age, giving a history of twenty years of suffering from urinary disease, consulted me first June 1, 1894. At that time he was suffering from inflammation of the bladder, the urine was ammoniacal, the prostate very much enlarged and he was using the catheter every four hours. He manifested the various phenomena of septic infection; he had suffered greatly from strangury and tenesmus, which had produced prolapse of the bowel; he had acquired the common habit of those suffering from urinary tension, due to enlarged prostate, of pinching and drawing up the penile organ until the prepuce was notably elongated. The pendulous urethral canal was so narrowed by the results of inflammatory action that a No. 8 American scale catheter could not be introduced without great difficulty. After prescribing appropriate internal treatment S. was taught this method of irrigation, which he learned under my personal supervision at his own home. My next procedure was enlargement of the urethral canal by internal urethrotomy, and S. was instructed to irrigate twice a day and to gradually discontinue the use of the catheter. As the urgent symptoms subsided the catheter was used less and less until it was employed but once a day for the purpose of drawing off residual urine in order that sleep might be uninterrupted. He steadily gained in his ability to pass water without a catheter, and after a few weeks was enabled to pass the night without being called up more than once. He returned to New York the first of September and wrote me that he was better than he had been in forty years. S. had begun catheter life and in spite of all that had been done for him was rapidly getting worse. This method of treatment saved him from a surgical procedure which might have been absolutely necessary to have saved his life. S. continues to use the catheter at bed time to draw off residual urine, but has no tenesmus or other urinary discomfort.

This process bears neither traumatism nor infection, it carries only good will. It decongests, it dehydrates, it cleanses, it purifies, it prevents and blanches inflammatory engorgements, it promotes absorption of inflammatory products. It leaves the full urethral space for voiding clots and other débris, it supplies abundant menstruum for flooding out during urination. There is no catheter wall to prevent fluids reaching and bearing medicament to the entire urethral surface. The patient himself can apply and carry out this treatment at his home—a great convenience for the doctor. Irrigation should not be used in a routine way. The disease or pathological condition should be diagnosticated and its cause sought and removed while symptoms are being palliated.

I have found this method of irrigation efficient in cleansing the urinary ways, maintaining and rendering the urinary secretions aseptic, mechanically useful in promoting urination and thus diminishing the use of the catheter. The catheter should be used only as a temporary makeshift. Its too frequent use causes impairment of the expulsive powers and its constant use for two years will permanently paralyse the powers of urination.

Diseases of the genito-urinary system of man have been neglected and their therapeutics are not well understood. Diseases of the genito-urinary system of woman have received marvelous surgical attention at the hands of our confrèrès, the gynecologists, while they, too, have neglected therapeutics.

If I have interested you and added something useful to you in your struggle with these maladies, I have accomplished all that I had hoped.

258 FRANKLIN STREET.

